# ADAR2 editing activity in newly diagnosed *versus* relapsed pediatric high-grade astrocytomas

**DOI:** 10.1186/1471-2407-13-255

**Published:** 2013-05-22

**Authors:** Sara Tomaselli, Federica Galeano, Luca Massimi, Concezio Di Rocco, Libero Lauriola, Angela Mastronuzzi, Franco Locatelli, Angela Gallo

**Affiliations:** 1 Laboratory of RNA Editing, Department of Pediatric Haematology/Oncology, Bambino Gesù Children’s Hospital, IRCCS, Piazza S. Onofrio 4, Rome 00165, Italy; 2 Pediatric Neurosurgery Department, Policlinico Gemelli, Largo A. Gemelli 8, Rome 00168, Italy; 3 Anatomopathology Department, Policlinico Gemelli, Largo A. Gemelli 8, Rome 00168, Italy; 4 Università di Pavia, Strada Nuova 65, Pavia 27100, Italy

**Keywords:** High-grade astrocytomas, RNA editing, ADAR2

## Abstract

**Background:**

High-grade (WHO grade III and IV) astrocytomas are aggressive malignant brain tumors affecting humans with a high risk of recurrence in both children and adults. To date, limited information is available on the genetic and molecular alterations important in the onset and progression of pediatric high-grade astrocytomas and, even less, on the prognostic factors that influence long-term outcome in children with recurrence. A-to-I RNA editing is an essential post-transcriptional mechanism that can alter the nucleotide sequence of several RNAs and is mediated by the ADAR enzymes. ADAR2 editing activity is particularly important in mammalian brain and is impaired in both adult and pediatric high-grade astrocytomas. Moreover, we have recently shown that the recovered ADAR2 activity in high-grade astrocytomas inhibits *in vivo* tumor growth. The aim of the present study is to investigate whether changes may occur in ADAR2-mediated RNA editing profiles of relapsed high-grade astrocytomas compared to their respective specimens collected at diagnosis, in four pediatric patients.

**Methods:**

Total RNAs extracted from all tumor samples and controls were tested for RNA editing levels (by direct sequencing on cDNA pools) and for *ADAR2* mRNA expression (by qRT-PCR).

**Results:**

A significant loss of ADAR2-editing activity was observed in the newly diagnosed and recurrent astrocytomas in comparison to normal brain. Surprisingly, we found a substantial rescue of ADAR2 editing activity in the relapsed tumor of the only patient showing prolonged survival.

**Conclusions:**

High-grade astrocytomas display a generalized loss of ADAR2-mediated RNA editing at both diagnosis and relapse. However, a peculiar Case, in complete remission of disease, displayed a total rescue of RNA editing at relapse, intriguingly suggesting ADAR2 activity/expression as a possible marker for long-term survival of patients with high-grade astrocytomas.

## Background

Astrocytoma grade III (anaplastic astrocytoma, AA) and astrocytoma grade IV (glioblastoma multiforme, GBM) are malignant, highly aggressive human brain tumors, characterized by an intrinsic tendency to recur. The median overall survival (OS) time after diagnosis is 12–18 months in both children and adults and decreases to a few months for patients with recurrence [1,2]. Despite multimodal treatment approaches, including extensive surgical resection and innovative radio- and chemotherapies, the outcome for patients with high-grade astrocytomas has not significantly improved over time. Of note, available data suggest that very young children (age <3 years) have a more favorable prognosis than older patients with similar tumors, even if recurrence is common also in this subset of patients [3].

Differently from adults in which malignant astrocytomas are the most frequent primary brain tumors, the pediatric counterparts account for only 6-12% of all brain neoplasms [4,5]. Consequently, to date limited information is available on the genetic and molecular alterations in pediatric patients important for the onset and progression of high-grade astrocytomas and even less is known about the prognostic factors that influence the long-term outcome in children with recurrence [5-7].

A-to-I RNA editing is an essential post-transcriptional mechanism that changes Adenosine (A) into Inosine (I) within RNA molecules due to the action of the ADAR (adenosine deaminase acting on dsRNA) enzymes. As Inosine is “read” as Guanosine by the splicing and translation machineries, the ADAR enzymes can generate a variety of RNAs and proteins different from those genetically coded. Three ADAR enzymes exist in mammals: ADAR1 and ADAR2 are ubiquitously expressed and catalytically active, whilst ADAR3 shows brain-specific expression and is enzymatically inactive [8,9]. ADARs can bind RNA targets through their RNA-binding domains (RBDs) and convert A into I thanks to the highly conserved deaminase domain (DM) [8,9]. RNA editing levels depend on the different substrates/sites, cell types, tissues and developmental stage [10,11]. *Adar1*^−/−^ and *Adar2*^−/−^ knockout mice die at embryonic or post-natal stages, respectively, indicating that these enzymes are essential for survival in mammals [12,13].

Compared to other tissues the mammalian brain carries the highest amount of inosines [14]. Indeed several edited transcripts have been identified in the central nervous system (CNS), where ADAR2 seems to play a major role [12]. Some transcripts, coding for proteins important for a correct brain development and function, undergo editing events that change amino acid sequence (recoding editing) in crucial positions for protein activity, such as the receptor subunits of the AMPA glutamate-gated ion channel (GluR-B, -C, -D), the Kainate receptors (GluR-5, GluR-6), the potassium voltage-gated channel (Kv1.1), the serotonin receptor (5-HT_2C_R) and the α3 subunit of the γ-amino butyric acid (GABA) receptor [9]. Interestingly, it has been shown that glutamate receptor antagonists inhibit *in vitro* proliferation of several human tumor cells, including gliomas [15] and that silencing of a specific AMPA receptor subunit reduces glioma growth *in vivo*[16]. Furthermore, it has been demonstrated that editing events within *GluR*-*B* inhibits glioma cell migration *in vivo*[17].

In view of these data, it is not surprising that alterations in A-to-I RNA editing in these transcripts have been observed in several human diseases affecting the CNS, including brain tumors [9]. In particular, a generalized hypoediting in both adult and pediatric high-grade astrocytomas when compared to normal brain tissues has been observed [9,18-21]. Moreover, we have recently demonstrated that the rescue of ADAR2 activity in astrocytoma cells prevents tumor growth *in vivo*, through the modulation of a specific molecular pathway involved in the cell cycle G1/S checkpoint [22].

The aim of the present study is to analyse ADAR2-mediated RNA editing profiles in four pediatric matched pairs of high-grade astrocytomas collected at the time of diagnosis and at recurrence, in order to investigate whether changes occur throughout disease progression.

## Methods

### Patients and samples collection

Four pediatric patients with high-grade astrocytomas, similar tumor location and local recurrences were enrolled in this study. The patients’ clinical data are summarized in Table 1.

**Table 1:** **Clinical features of four children with high**-**grade astrocytoma**

	**Case 1**	**Case 2**	**Case 3**	**Case 4**
**Sex**	F	M	M	F
**Age at diagnosis**	≤ 13 years	≤ 12 years	≤ 8 years	≤ 3 years
*Newly diagnosed tumor*				
**Tumor location**	P left	F left	FTP right	FP left
**Resection**	GTR	GTR	GTR	GTR
**Histology**	GBM	GBM	AA	GBM
**Ki**-**67** (**IHC**)	>10%	50%	7-10%	60%
**Pre**-**radiation CT**	/	/	/	infant protocol (*)
**RT doses**	54 Gys plus TMZ	54 Gys plus TMZ	54 Gys plus TMZ	59 Gys (at time of 3 year old)
**Post**-**radiation CT**	TMZ (6 courses)	TMZ (6 courses)	TMZ (6 courses)	/
*Recurrent tumor*				
**DFS** (**months**)	8	14	33	22
**Recurrence**	local	local	local	local
**Resection**	GTR	GTR	GTR	GTR
**Histology**	GBM	GBM	AA	GBM
**Ki**-**67** (**IHC**)	40%	50%	60%	50%
**Adjuvant CT**	TMZ /PCV (1 course)°	TMZ /PCV (4 courses)°	TMZ /PCV (6 courses)°	TMZ /irinotecan (12 courses)
**Outcome**	dead	dead	dead	alive
**LPS score**	/	/	/	90
**Disease**	/	/	/	CR; off therapy
**OS** (**months**)	10	26	40	57

*P* Parietal, *F* Frontal, *FTP* Fronto-temporo-parietal, *FP* Fronto-parietal, *GTR* Gross Total Resection, *GBM* Glioblastoma, *AA* Anaplastic Astrocytoma, *IHC* immunohistochemistry, *RT* Radiotherapy, *CT* Chemotherapy, *TMZ* Temozolomide, *DFS* Disease Free Survival, *PCV* Procarbazine-Lomustine-Vincristine, *LPS score* Lansky performance score (from 100 to 0, with 100= healthy status), *CR* Complete Remission, *OS* Overall Survival.

°Until progression and death.

*Infant protocol according to the National Therapeutic Indications for infant with GBM: Methotrexate and Vincristine (1 course), Etoposide (1 course), cyclophosphamide and Vincristine (1 course), thiotepa (2 courses) followed by stem cell auto-grafting.

The matched tumor samples were dissected from the proliferative core of the tumors and split in two halves, with one half fixed in 10% formalin for immunohistochemistry (IHC) analysis and the second half stored at −80°C for molecular studies. Non-tumoral white matter samples (a pool of two), isolated from the same brain area of the tumors and obtained from pediatric patients undergoing focal brain resection for head injury sequelae (e.g. brain contusion), were used as normal control after being anonymized.

The study was revised and approved by the Institutional Review Board (IRB) of the local committee (Bambino Gesù Children’s Hospital, Rome) on the use of human samples for experimental studies. Informed consent was obtained from all the patients’ parents to the use of biological samples for research purposes.

### Editing analysis

For RNA editing analysis, total RNA was isolated from tumor and control brain tissues with TRIzol reagent (Invitrogen, Carlsbad, CA, USA) according to the manufacturer’s instructions. Total RNAs were treated with DNAse and cDNAs were generated using the ImProm-II Reverse Transcription System (Promega, Madison, WI, USA) and random hexamers or transcript-specific oligonucleotides (available on request). Three independent RT-PCRs (reverse transcriptase-polymerase chain reactions) were performed for each sample. Direct sequencing (ABI 3500 Genetic Analyzer, Applied Biosystems, Foster City, USA) was performed on cDNA pools and the editing levels at specific sites were measured as previously described [20,23]. Briefly, in the sequence chromatogram the Adenosine nucleotide that undergoes editing appears as a double peak: Adenosine for the unedited forms and Guanosine for those edited (the height of the two peaks was used for calculation of editing percentage) (Additional file 1: Figure S1).

### Analysis of mRNA expression levels

Gene-specific exon-exon boundary PCR products (TaqMan gene expression assays, Applied Biosystems) were measured by means of a PE Applied Biosystems PRISM 7700 sequence detection system during 40 cycles. β-*actin* mRNA was used for normalization and relative quantification of gene expression according to the 2^-ΔΔCt^ method. Real-time assays were repeated in triplicates from two independent RT-PCRs. The primers were supplied by Applied Biosystems: ADAR2, ID Hs00953730_m1; β-actin, ID Hs99999903_m1. The expression level of each recurrence was calculated as relative-fold increase compared to that of the corresponding newly diagnosed tumor arbitrarily set to 1.

To test *Ki*-*67* expression levels, we performed semi-quantitative RT-PCRs directly on the total RNA isolated from tumor and control tissues. β-*actin* was used to normalize the RT-PCR reactions. Ki-67 levels were also evaluated by IHC on the paraffin-embedded tissues by two independent experienced neuropathologists.

## Results

### RNA editing in newly diagnosed *versus* recurrent pediatric high-grade astrocytomas

It is emerging the idea that differences in molecular characteristics can be present in newly diagnosed *versus* recurrent malignant high-grade astrocytomas [2,24]. We therefore investigated whether ADAR2-mediated RNA editing, found to be important in astrocytomas, may vary throughout disease progression in four pediatric patients with supratentorial recurrent high-grade astrocytomas (Table 1).

We focused on recoding editing events of transcripts that translate into brain membrane receptors or ion channels, such as the receptor subunit of the AMPA channel (GluR-B), the receptor subunits of the Kainate channel (GluR-5 and GluR-6) and the potassium channel (Kv1.1), because these sites are mainly, if not exclusively, edited by ADAR2 enzyme [12].

We analyzed editing levels of the *GluR*-*B* transcript at the Q/R and the R/G sites, the *GluR*-*6* transcript at three recoded positions identified as the I/V, Y/C and Q/R sites, the *GluR*-*5* transcript carrying the Q/R edited site and the *Kv1*.*1* transcript carrying the I/V edited site. Editing levels at all these sites were also tested in normal white matter tissues used as control and dissected from the same area of the brain where the tumors developed.

**Figure 1:**
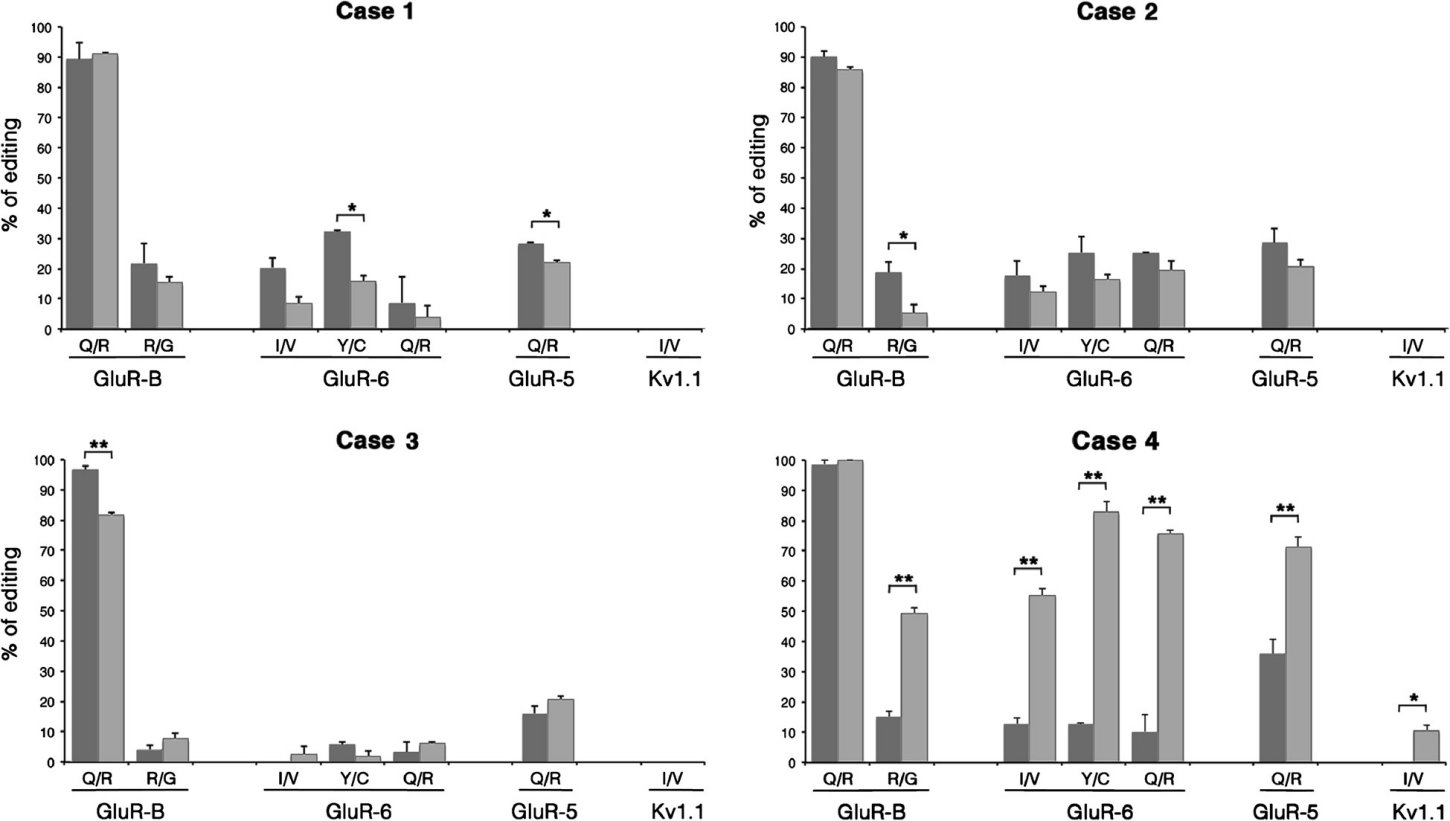
**RNA editing analysis in Cases 1–4.** RNA editing levels at the *GluR-B* Q/R and R/G sites, *GluR-6* I/V, Y/C and Q/R sites, *GluR-5* Q/R site and *Kv1.1* I/V site were analyzed in newly diagnosed (dark gray) and recurrent (light gray) high-grade astrocytomas in Cases 1 to 4. The editing values are expressed as a percentage of the mean of three independent experiments. *Error bars* indicate standard error of the mean (S.E.M.), *p<0.05, **p<0.01.

RNA editing analysis of tumor samples at diagnosis showed a significant loss of ADAR2 activity when compared with control tissues at all the sites analyzed (Table 2), as expected from previous studies [19-21]. Additionally, when we compared the editing profiles of newly diagnosed tumors with the corresponding relapses, we observed a generalized further loss of editing levels, with some editing sites showing a statistically significant decrease in the relapsed tumors compared with the previous lesions: the *GluR-6* Y/C site (p≤0.05) and the *GluR-5* Q/R site (p≤0.05) of Case 1, the *GluR-B* R/G site (p≤0.05) of Case 2 and the *GluR-B* Q/R site (p≤0.01) of Case 3 (Figure 1 and Table 2).

**Table 2:** ADAR2 edited sites and their relative percentage of editing

**ADAR2 edited sites**	**WM**	**Case 1**		**Case 2**		**Case 3**		**Case 4**	
**(% of editing ± s.e.m.)**	**(Ctrls)**								
		**N**	**R**	**N**	**R**	**N**	**R**	**N**	**R**
**GluR-B Q/R**	100 (±0)	90.3 (±5.6)	92.1 (±0.3)	87.6 (±1.8)	83.5 (±0.9)	98.15 (±1.1)	83 (±0.7)	98.7 (±1.3)	100 (±0)
**R/G**	53.2 (±4.2)	21.9 (±6.5)	15.4 (±2)	18.1 (±3.2)	5 (±2.7)	4.4 (±1.4)	7.9 (±1.9)	15.1 (±1.8)	49.3 (±1.8)
**GluR-6 I/V**	58.1 (±0.7)	20.5 (±3.3)	8.3 (±2.5)	17.1 (±4.8)	12 (±1.5)	0 (±0)	2.7 (±2.7)	12.9 (±1.7)	55.2 (±2.4)
**Y/C**	73.5 (±6.9)	32.4 (±0.45)	15.7 (±2)	24.6 (±5.1)	15.7 (±1.8)	6.1 (±0.8)	1.9 (±1.9)	12.7 (±0.3)	82.8 (±3.5)
**Q/R**	74.6 (±0.9)	8.6 (±8.6)	3.8 (±3.8)	24.5 (±0.1)	19 (±2.7)	3.4 (±3.4)	6.4 (±0.5)	10.3 (±5.3)	75.9 (±0.9)
**GluR-5 Q/R**	63.8 (±1)	28.7 (±0.2)	22 (±0.9)	27.6 (±4.6)	20 (±2.2)	16.4 (±2.4)	21.1 (±1.2)	35.9 (±4.9)	71.5 (±3)
**Kv1.1 I/V**	9.6 (±2)	0 (±0)	0 (±0)	0 (±0)	0 (±0)	0 (±0)	0 (±0)	0 (±0)	10.7 (±1.7)

*WM* white matter, *N* newly diagnosed tumor, *R* recurrent tumor.

% of editing is expressed as mean of three independent experiments ± S.E.M.

Unexpectedly, the recurrence of the youngest patient (Case 4, age at diagnosis ≤ 3 years; Table 1) displayed a completely different RNA editing profile in comparison to the tumor at diagnosis, showing significantly higher editing levels at all the analyzed sites (Figure 1 and Table 2).

### *In vivo* rescue of ADAR2 RNA editing activity

Considering the surprising results observed in the recurrence of Case 4, we decided to analyze in this patient additional recoding editing sites previously found to be edited, mainly or partially, by ADAR2. We performed RNA editing analysis of the *Gabra*-*3* I/M site (edited by both ADAR1 and ADAR2) [25], the *BLCAP* Y/C, Q/R sites (edited by both ADAR enzymes) and the K/R site (edited mainly by ADAR2) [26,27] in the tumor tissues of Case 4 and controls.

Editing within the *Gabra*-*3* transcript controls trafficking of α3-containing receptors to the cell membrane [28]. Despite the fact that the role of editing events within *BLCAP* are still unknown, it has been proposed that this protein is a novel prognostic biomarker in bladder cancer and it is associated with cell proliferation [29].

**Figure 2:**
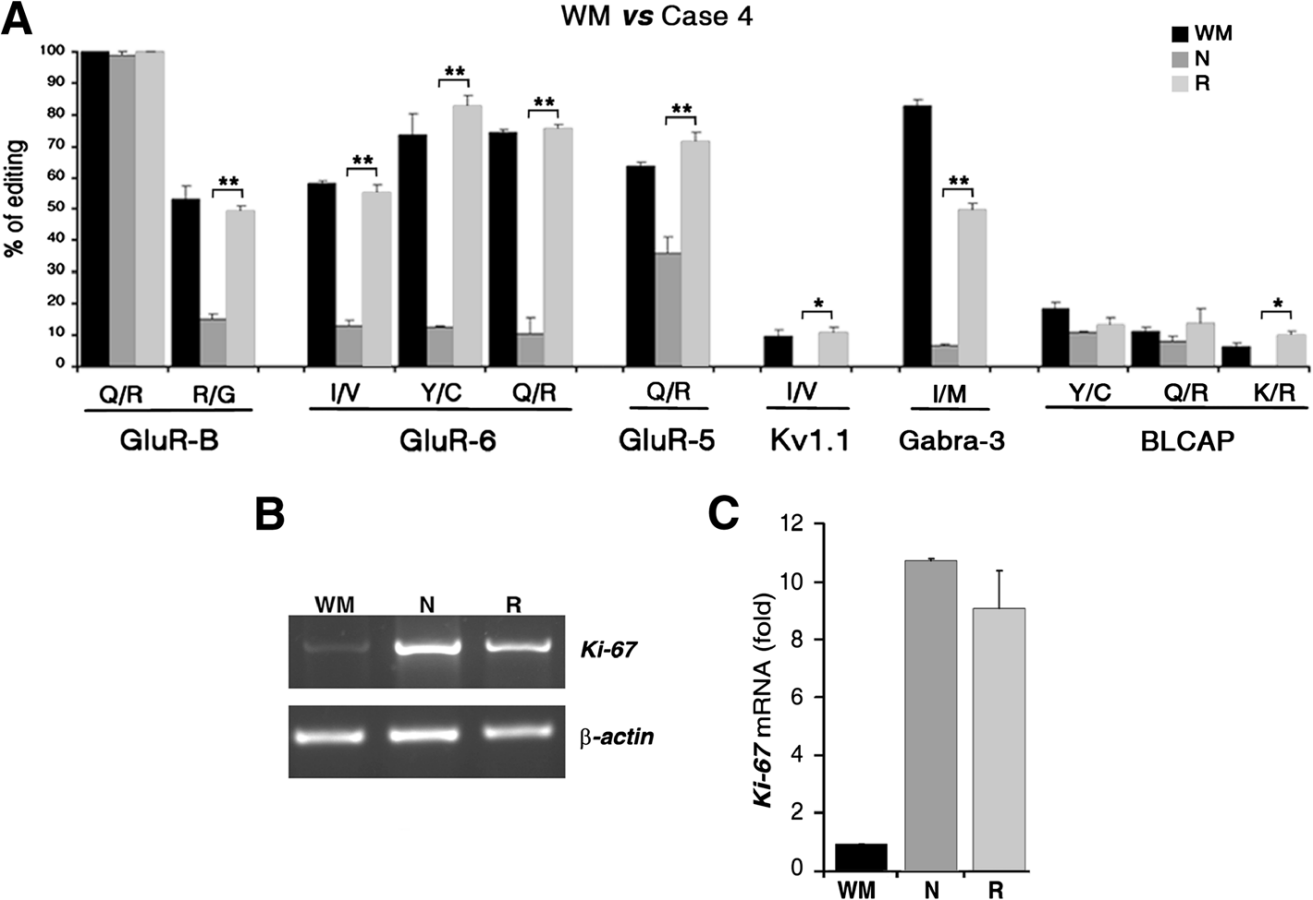
**Comparative molecular analysis in Case 4 *versus *control.** (**A**) Comparative analysis of the RNA editing levels at the *GluR-B* Q/R and R/G sites, *GluR-6* I/V, Y/C and Q/R sites, *GluR-5* Q/R site, *Kv1.1* I/V, *Gabra*-3 I/M site and *BLCAP* Y/C, Q/R and K/R sites among normal white matter (WM, black), newly diagnosed (N, dark gray) and recurrent (R, light gray) tumor tissues of Case 4. The editing values are expressed as a percentage of the mean of three independent experiments. *Error bars* indicate standard error of the mean (S.E.M.), *p<0.05, **p<0.01. (**B**) A representative example of *Ki*-*67* mRNA expression levels analysed by semi-quantitative RT-PCRs in the control (normal white matter), newly diagnosed and recurrent tumor samples of Case 4. (**C**) Densitometric analysis of *Ki*-*67* mRNA expression is represented in arbitrary units calculated as a relative-fold increase in expression compared to the control arbitrarily set to 1. Each sample was normalized to β-*actin* mRNA. Error bars indicate standard error of the mean (S.E.M.) (n=3).

This further analysis confirmed a rescue of RNA editing levels in the relapse of Case 4 for all the tested sites, with editing values similar to those found in normal brain (Figure 2A). Of note, the only site of *BLCAP* transcript showing a significant editing rescue was the K/R site, which is the only one mainly modified by ADAR2 [26].

In order to rule out any possible unintentional contamination of non-tumor tissue in the relapse of Case 4, we measured the levels of *Ki*-*67* cell proliferation index directly on the RNA samples used for the RNA editing molecular assays (Figure 2A). As expected for neoplastic tissues, both the newly diagnosed and recurrent tumor samples of Case 4 showed over-expression of *Ki*-*67* mRNA when compared with normal white matter (Figure 2B-C). A similar result on the same samples was obtained by IHC analysis (Table 1). High *Ki*-*67* levels were also detected by semi-quantitative RT-PCR (data not shown) and IHC (Table 1) in the tumor tissues of Cases 1-2-3.

### *ADAR2* expression levels in pediatric high-grade astrocytomas

ADAR2 is the enzyme mainly responsible for the recoding editing at the sites analyzed in this study [12,20]. Therefore, we investigated whether fluctuation in *ADAR2* mRNA occurred in tumor samples that may partially explain the editing profiles of the all Cases reported.

**Figure 3:**
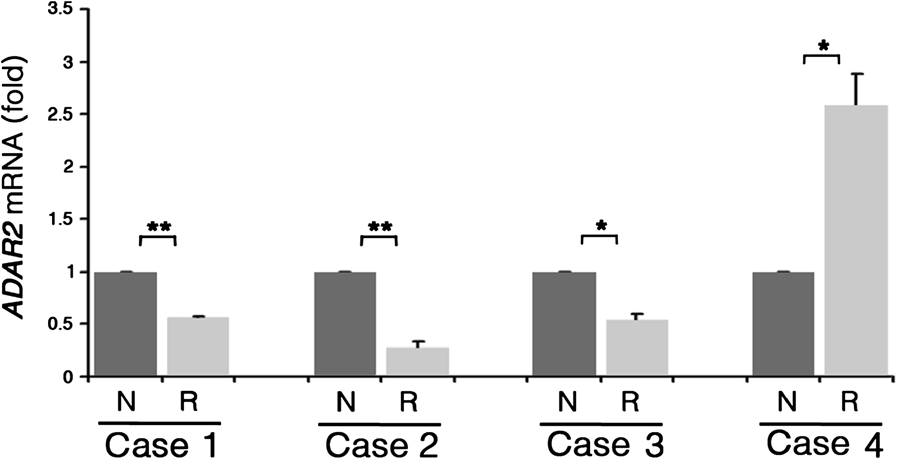
**ADAR2 expression levels in Cases 1–4.** qRT-PCR analysis of *ADAR2* mRNA from newly diagnosed (dark gray) and recurrent (light gray) tumors in Cases 1 to 4. The expression levels of each recurrence were calculated as a relative-fold increase compared to the corresponding newly diagnosed tumor arbitrarily set to 1. Each sample was normalized to β-*actin* mRNA. Mean ± s.d. (n=2), *p<0.05, **p<0.01.

We found a significant decrease of *ADAR2* expression in the recurrences of Cases 1–3 when compared to their newly diagnosed tumors (Figure 3). On the contrary, a significant higher *ADAR2* expression level was found in the relapse of Case 4 when compared with the tumor at diagnosis (Figure 3), which can correlate with the rescued editing profiles found in the recurrence of this patient (Figure 2A).

## Discussion

High-grade astrocytomas are very aggressive brain tumors, with GBM (or astrocytoma grade IV) being one of the most lethal tumors in humans. Despite the novel and aggressive surgical/therapeutic approaches, after a short period of remission these tumors frequently recur, with a median survival, after recurrence, of only few months [2]. The molecular mechanisms involved in the formation of malignant astrocytomas and their subsequent recurrences, as well as the signature associated with long-term survival and a positive outcome are still poorly known, especially in children, due to the rarity of these tumors during the pediatric age [6,24,30].

RNA editing is an essential genetic recoding process that enhances the molecular diversity of RNAs and proteins at post-transcriptional level to different extents, depending on the cell types and tissues. In particular, ADAR2-mediated RNA editing is essential for the functional activity of many proteins expressed in the CNS from fly to mammals [9].

It has been shown that astrocytomas are characterized by a general decrease of RNA editing mediated by ADAR2 enzyme [19-22,26] and that a correlation exists between the progressive loss of ADAR2 activity and the increasing grade of tumor, with the lowest editing levels found in AAs and GBMs [20]. Furthermore, we have recently demonstrated that a recovery of ADAR2 editing activity in astrocytoma cells is necessary and sufficient to significantly inhibit tumor growth in a mouse model [22].

Considering the above findings, we investigated whether differences exist in RNA editing profiles mediated by ADAR2 between malignant high-grade astrocytomas at initial presentation and their subsequent relapse in the same patient (Table 1). To the best of our knowledge, this is the first comparative report of RNA editing analysis performed on matched pairs of newly diagnosed and recurrent tumor tissues in the same patient.

The small size of patient cohort analyzed in this study is mainly due to the rarity of high-grade astrocytomas in children, together to the difficulty in collecting tumor samples from the same patient both at diagnosis and at recurrence. Additionally, as RNA editing profiles change depending on different brain areas [20,31], we needed to collect tumor samples developed within the same brain region (supratentorial astrocytomas) from different patients.

We found an overall general decrease in RNA editing levels in both newly diagnosed and relapsed tumors in 3 out of 4 cases when compared with controls (Figure 1 and Table 2), with a significant further drop of editing in the recurrences only at few specific editing sites (Figure 1 and Table 2).

These results suggest that ADAR2-mediated RNA editing, at least on the re-coding editing sites analyzed herein, is a molecular signature for high-grade astrocytomas that does not dramatically change during tumor recurrence in children.

The most surprising result was the editing profile of Case 4, the only surviving patient (Table 1). As compared to diagnosis, its relapse sample showed a recovery of RNA editing levels at all the sites tested, with values resembling those observed in control white matter dissected from the same brain area where the tumor developed (Figure 2A). These findings were unexpected, considering that previous studies in adult and pediatric astrocytomas always reported a significant editing decrease in high-grade astrocytomas [19-22].

Editing activity does not always correlate with mRNA or protein expression of ADAR enzymes [11]. According to this, a recent study showed the existence of “mediators” (i.e. proteins) that can modulate ADAR2 efficiency [32]. Nevertheless, we decided to test *ADAR2* expression by qRT-PCR in our patients and only in Case 4 we found a significant increase of *ADAR2* in the relapse compared to the newly diagnosed tumor (Figure 3). This finding correlates with the rescued editing profiles observed in the Case 4 relapsed tumor (Figure 2A). Notably, we have recently demonstrated that the forced expression of the active ADAR2 enzyme in astrocytoma cells rescues editing levels at specific sites (such as the ones tested here) and that, most importantly, this editing rescue is able to inhibit tumor growth with a significantly prolonged overall survival of mice injected with tumor cells overexpressing ADAR2 [22].

At present, little is known regarding the physiological regulation of *ADAR2* expression, however it has been shown that both its expression and activity are markedly enhanced in response to glucose in pancreatic islets and beta-cells [33]. Moreover, it has been shown that in neuronal cells the cAMP response binding element (CREB), an important transcription factor, can induce ADAR2 expression [34]. The observation that infants follow a different protocol than older children (Table 1) is intriguing and suggest that ADAR2 expression and/or RNA editing levels could be recovered in this particular subset of patients, possibly due to specific treatments or drugs. Considering the findings of *ADAR2* upregulation in a peculiar Case (Case 4), we asked whether a possible correlation exist between *ADAR2* mRNA expression and pediatric patient survival, interrogating available datasets. We found only a glioma array specific for pediatric patients (but not for infant) in which the clinical outcome was also reported (http://r2.amc.nl, dataset Paugh-53-MAS5.0-u133p2). We observed that, at least in this dataset, there is not a statistically significant correlation between *ADAR2* levels and outcome, even if a slight decrease of *ADAR2* expression is reported for patients died of disease compared to patients alive (data not shown).

Currently, total tumor resection, aggressive treatment and diagnosis at a younger age have been associated with longer survival of pediatric patients with high-grade astrocytomas [6,35]. Thus, it is intriguing to speculate that in very young children high-grade astrocytomas may be biologically different [3,36]. The hypothesis that younger patients (as in the Case 4 reported here) might be able to recover ADAR2 expression/activity, due to still unknown endogenous cellular factors or maybe induced by specific treatments or drugs, deserves additional investigations.

Furthermore, it would be worth considering the role of ADAR2 activity/expression as possible marker for long-term survival of patients with recurrent high-grade astrocytomas.

## Conclusions

Despite the low number of paired samples investigated, RNA editing mediated by ADAR2 seems to further decrease significantly only at few specific sites throughout disease progression. Moreover, our findings relative to one peculiar Case (age ≤ 3 years) suggest that ADAR2 activity can be rescued *in vivo* in tumor cells, raising the intriguing possibility that editing recovery may have contribute to the favorable outcome of this patient, as suggested by mouse model studies (22).

## Competing interests

The authors declare that neither financial nor non-financial competing interests exist.

## Authors’ contributions

ST and FG carried out the molecular genetic studies and drafted the manuscript. LM, CDR, FL and AM provided the tumor samples as well as the clinical details of the patients and revised the manuscript. LL performed the histological analysis of the tumor samples. AG designed the study, analyzed the data and wrote the manuscript. All authors read and approved the final manuscript.

## Pre-publication history

The pre-publication history for this paper can be accessed here:

http://www.biomedcentral.com/1471-2407/13/255/prepub

## Supplementary Material

Additional file 1: Figure S1 Editing levels of *GluR*-*5* substrate in control brain tissue and Case 4. Sequence chromatograms of *GluR*-*5* substrate using RNA extracted from control white matter (WM), Case 4 newly diagnosed GBM (N) and recurrence (R). The Q/R edited site is represented as a double peak (adenosine plus guanosine) and is indicated by arrows. Click here for file
