# Supplementary material for: ADAR2 editing activity in newly diagnosed versus relapsed pediatric high-grade astrocytomas
Source: BMC Cancer. 2013 May 22;13:255. doi: 10.1186/1471-2407-13-255 (PMC3663793; doi:10.1186/1471-2407-13-255)
Supplement: Additional file 1: Figure S1 — Editing levels of GluR-5 substrate in control brain tissue and Case 4. Sequence chromatograms of GluR-5 substrate using RNA extracted from control white matter (WM), Case 4 newly diagnosed GBM (N) and recurrence (R). The Q/R edited site is represented as a double peak (adenosine plus guanosine) and is indicated by arrows. [file 1471-2407-13-255-S1.pdf]

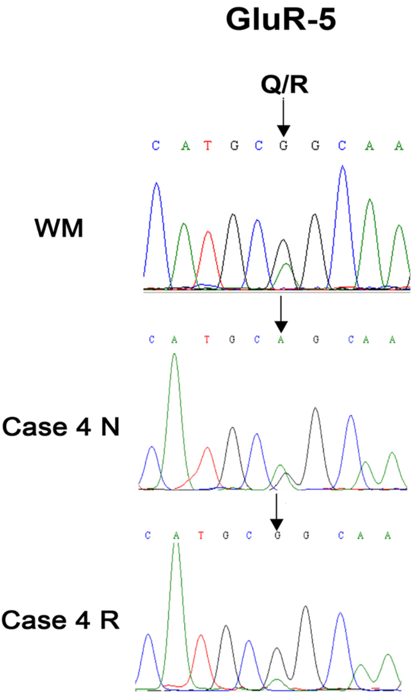

Figure S1. Editing levels of *GluR-5* substrate in control brain tissue and Case 4.

Sequence chromatograms of *GluR-5* substrate using RNA extracted from control white matter (WM), Case 4 newly diagnosed GBM (N) and recurrence (R). The Q/R edited site is represented as a double peak (adenosine plus guanosine) and is indicated by arrows.
